# Anti-Cytokine Autoantibodies in Systemic Lupus Erythematosus

**DOI:** 10.3390/cells9010072

**Published:** 2019-12-27

**Authors:** Hwee Siew Howe, Bernard Pui Lam Leung

**Affiliations:** 1 Department of Rheumatology, Allergy and Immunology, Tan Tock Seng Hospital, Singapore 308433, Singapore; Bernard.Leung@SingaporeTech.edu.sg; 2 Singapore Institute of Technology, Singapore 138683, Singapore

**Keywords:** autoantibodies, cytokines, autoimmunity, systemic lupus erythematosus

## Abstract

Cytokine dysregulation is characteristic of systemic lupus erythematosus (SLE), a systemic autoimmune disease of considerable heterogeneity. Insights gained about the cytokine dysregulation in SLE have the potential for identifying patient subsets before the onset of clinical disease and during established disease. Clustering patients by cytokine and disease activity subsets is more informative than isolated cytokine studies, as both pro inflammatory and immunoregulatory cytokines contribute to the cytokine dysregulated state in SLE. Endogenous anti-cytokine autoantibodies (ACAAs) may be involved in the regulation of cytokine biology by reducing excessive production or by prolonging their half-life in the circulation through the formation of cytokine-antibody immune complexes. Although endogenous ACAAs may have deleterious effects such as contributing to immunodeficiency states, their role in the pathophysiology of autoimmune conditions such as SLE has yet to be clearly elucidated. The aim of the present article is to provide a focused review of the current knowledge of ACAAs in SLE.

## 1. Cytokines in Systemic Lupus Erythematosus (SLE): Insights Gained

### 1.1. Cytokine Disturbances Precede Clinical Disease in SLE

Two studies have demonstrated that immune dysregulation precedes the development of clinical disease in SLE [1,2]. Elevated levels of cytokines reflecting both innate and adaptive immune system activation including IL-6 (Th2/Th17/Tfh), IL-12 p70, and interferon–gamma (Th1), IL-4, and IL-5 (Th2) as well as the chemokine interferon (IFN) gamma inducible protein 10(IP-10) have been found in patients more than three years before they satisfied SLE classification criteria. Conversely, levels of the regulatory mediator TGF-beta (TGF-β) were significantly lower. As they neared a classifiable disease, they developed dysregulation of multiple tumour necrosis factor (TNF) superfamily cytokines including TNFRI, TNFRII, Blys, and APRIL). Expansion of SLE associated autoantibody specificities (anti-Ro first, then RNP, Sm, dsDNA, and La) began significantly later than the onset of dysregulation of IL-4, IL-5, IL-6, IFN-γ, and MIG, as SLE patients evolved into classifiable disease. The combination of soluble mediator levels and anti-nuclear antibody (ANA) positivity increased the accuracy of the prediction of developing SLE compared to ANA positivity alone and to IL-5 and IL-6 levels (84%, 58.55, and 79%, respectively). Nephritis was more likely to develop in patients who were ANA negative with elevated levels of IL-5 and IL-6. Thus, there may be potential for further exploration into these differences in cytokine dysregulation in combination with autoantibody profiles to be developed into tools for the prediction of clinical phenotypes and disease course. In future, there is also the possibility of modulating the cytokine dysregulation to prevent the development of clinical disease, using agents with a low risk of toxicity such as hydroxychloroquine or cytokine specific immune modifiers.

### 1.2. Clustering Patients by Cytokine and Disease Activity Subsets Is More Informative Than Isolated Cytokine Studies, as Both Pro Inflammatory and Immunoregulatory Cytokines Contribute to the Cytokine Dysregulated State in SLE

Immune dysfunction arising from cytokine dysregulation culminates in tissue inflammation and organ damage. There has been significant variability in findings of cytokine studies in SLE due to differences between populations and the methodologies. In general, both inflammatory cytokines such as type I and type II interferons (IFNs), interleukin-6 (IL-6), IL-1, tumor necrosis factor-alpha (TNF-α), and immunoregulatory cytokines including IL-10 and transforming growth factor-beta (TGF-β) contribute to the cytokine dysregulated state in SLE [3,4]. In particular, the type 1 interferon (IFN) pathway is well established to be dysregulated in SLE. The IFN gene signature, an upregulated group of type 1 IFN responsive genes, is found in the peripheral blood in over 50% of adult and the majority of pediatric patients [5]. Two recent studies described below provide a better understanding of cytokine levels and their correlations with disease activity and phenotype in SLE.

Serum levels of isolated cytokines do not reflect the underlying complex immune interactions in disease pathogenesis. To address this, Reynolds et al. performed multiplex high-sensitivity based enzyme-linked immunosorbent assay (ELISA) with a panel of 10 cytokines relevant to SLE [6]. Cytokine grouping was established based on a principal cellular source relevant to disease pathogenesis, namely monocytes and innate cells (IFNα, IL-10, IL-18, C-X-C-motif chemokine ligand (CXCL10), monocyte chemotactic protein (MCP-1), pentraxin related protein (PTX3)), B cells (B lymphocyte stimulator (BLyS), CXCL13), and T cells (IL-10, IL-17, IL-21). Interestingly, all 10 biomarkers were measurable in most patients, and with the exception of IL-17, all cytokines were quantifiable in over 80% of SLE samples. Based on cluster analysis, patients could be further divided into three distinctive subsets. Groups 1 and 2 with active disease were segregated by their unique cytokine clusters with raised IL-10, IL-17, IL-21, BLyS, and IFNα and association with smoking in Group 1; while in contrast, raised CXCL10, CXCL13, and association with low complement C3 and C4, and raised dsDNA titers were features of Group 2. Furthermore, IL-18 was strongly increased in both groups. In contrast, all cytokines were lower than in Groups 1 and 2 in the Group 3 patients with inactive disease. Contrary to these findings, the efficacy of belimumab (a fully humanized IgG1 monoclonal antibody directed against soluble BLyS) in clinical trials has been correlated with hypocomplementemia and raised anti-dsDNA antibody titers, features of Group 2 with lower BLyS levels [7]. As the subjects were predominantly Caucasian, further validation of these findings is required in different ethnic populations and in larger cohorts.

Another recent study examined cytokine profiles in clinically quiescent SLE patients who had been off treatment for two years or more [8]. Cytokines and chemokines produced by stimulated monocytes (IL-1α, IL-1β, IL-6, IL-10, IL-12 p40, IL-12 p70, IL-23, TNF-α), proinflammatory T cell subsets (IL-2, IFN-γ, IL-17, IL-21), and those produced and/or induced by IFN-α (IFN-α, granulocyte-macrophage colony stimulating factor (GM-CSF), MCP-1, MCP-2, interferon gamma-induced protein 10 (also known as CXCL10), chemokine (C-C motif) ligand 5 (CCL5), and TNF-related apoptosis-inducing ligand (TRAIL) were selected. The gene expression profile (IFN-5 score) of five IFN-induced genes (OAS1, IFIT1, MX1, LY6E, and ISG15) was also investigated [8]. Serologically active (with hypocomplementemia and raised anti-dsDNA antibody titers) clinically quiescent (SACQ) patients demonstrated a reduced production of proinflammatory cytokines, despite the persistence of autoantibodies and hypocomplementemia. In both serologically and clinically active (SACA) and SACQ patients, levels of anti-dsDNA antibodies or complement C3 did not correlate with the IFN-5 score. The authors concluded that the production of proinflammatory cytokines and chemokines in SACQ patients was inhibited despite the presence of immune complexes by undefined immunologic mechanisms, possibly including the activity of anti-cytokine autoantibodies (ACAAs). 

### 1.3. Serum IFN-Regulated Chemokine Levels Correlate Best with Disease Activity

IFN, with its broad range of effects on the immune system, plays a critical role in SLE pathogenesis, with immune complexes inducing the overproduction of IFN-α by pDCs. Increased expression of IFN-regulated genes (the IFN gene signature) in the peripheral blood has been associated with increased severity of SLE disease [9]. However, although the interferon signature metric (ISM) is known to be associated with serological activity including hypocomplementemia and raised titers of anti-dsDNA antibodies, antibody positivity to extractable nuclear antigens (ENA), and raised serum BAFF, the ISM itself is not correlated with clinical disease activity. The presence of elevated serum levels of IFN-α ranged from 5% to 70% of SLE patients [10,11], the lower prevalence possibly arose from factors affecting their detection by ELISA such as blocking or auto-antibody interactions.

B-cell activating factor (BAFF), also known as BLyS, a member of the TNF superfamily of cytokines, contributes to SLE disease pathogenesis through promoting the survival of autoreactive B-cells, allowing their escape from negative selection [12,13]. Raised titers of anti-dsDNA and anti-Sm antibodies, hypocomplementemia, and increased BAFF levels are an independent risk factor for SLE disease flares [7]. Despite the strong correlation of BAFF levels with SLE activity, it has been suggested that elevated serum BAFF may reflect B cell activation, rather than act as a driver of inflammation in SLE [14]. Recent work by Sjöstrand et al. showed that type I IFNs drive the expression of BAFF through the binding of interferon responsive factors (IRFs) to a novel interferon responsive element (ISRE) site in the BAFF promoter [15]. Thus, type I IFN blockade could potentially downregulate BAFF, with a consequent reduction of autoreactive B cell clones and autoantibodies.

In contrast to the variable serum levels of interferon, serum IFN-regulated chemokines correlate well with lupus activity, in parallel with disease flare and remission [16,17,18]. In a longitudinal analysis of 267 SLE patients over a one year follow up, Bauer et al. [16] found that serum levels of the IFN regulated chemokines CCL2 (MCP-1), CCL19 (macrophage inflammatory protein, MIP-3B), and interferon gamma-inducible protein -10(IP-10) significantly outperformed standard serological tests. IP-10 in particular, was consistently the chemokine most strongly associated with current and future disease activity. This was a finding also shared by two studies in Asian lupus patients [19,20]. In particular, we observed elevated serum IP-10 levels in our SLE patients, which were significantly higher in the presence of active hematological and mucocutaneous manifestations. Serial IP-10 levels correlated with longitudinal change in SLE activity, even at low levels where anti-dsDNA antibody and complement levels remain unchanged, and may represent a more sensitive marker for monitoring disease activity than standard serological tests [20].

## 2. Anti-Cytokine Autoantibodies (ACAAs)

Endogenous ACAAs are found in the circulation and may mediate diverse immunological functions depending on their specific interaction with the cytokine. For an ACAA to be deemed of biological relevance, saturable binding to the recombinant cytokine in assays as well as substantial specific binding of the cytokine in vivo needs to be demonstrated [21,22]. In addition to concentration, epitope specificity, avidity, isotype, and subclass influence the capacity of autoantibodies for neutralizing their cognate cytokine. Significant physiological and pathological effects that occur with high levels (nanomolar concentrations) of ACAAs neutralizing ACAAs decrease the bioavailability of cytokines by inhibiting binding to their cognate receptors; reducing the activity of the cytokine [22]. It is also possible that ACAAs may enhance and prolong cytokine activity in cytokine–anticytokine antibody immune complexes that interact with various immuno-stimulatory receptors. Cytokine/autoantibody immune complexes in the circulation exist in equilibrium with their free cytokine and free autoantibody in concentrations that vary with the levels of cytokine to be neutralized. 

### 2.1. ACAAs in Healthy Individuals

Autoantibodies are generated when there is a breakdown of central and/peripheral tolerance. Mechanisms include insufficient thymic expression of target self antigens with the escape of autoreactive T cells, and repeated stimulation by these target self antigens in the periphery [22]. Lemos Rieper et al. proposed that when there is highly stimulated cytokine gene transcription, alternative forms of cytokines are produced that render them highly immunogenic [21], and this could be influenced by environmental and genetic factors, infection, drugs, neoplasms, and aging. ACAAs are present in most healthy individuals, where they may have a biological role in regulating cytokine activity, whether through neutralization or prolongation of the action of the cytokines as above-mentioned. Estimates of their true prevalence have varied due to the different methods of detection employed between studies. Their ubiquitous presence has been demonstrated in healthy individuals, with 100 percent having autoantibodies to IL-2, IL-8, TNF-α, vascular endothelial growth factor (VEGF), and granulocyte-colony stimulating factor (GCSF); 93.3 percent to IL-4; 73.3 percent to IL-10; and 6.7 percent to IL-6 and IFN γ in an earlier study [23]. 

In addition, human immunoglobulin products have been shown to contain ACAAs with substantial saturable binding to several cytokines including IL-1α, type I interferons, IL-6, IL-10, and GM-CSF [21,22]. A study of Danish blood donors found that 20% had detectable IgG binding to IL-6; 10% had specific saturable high avidity IgG binding to IL-6; 1% had substantial binding; and 0.1% had levels that resulted in IL-6 deficiency [24]. ACAAs to IL-1α [25], TNFα [26], IL-6 [27], and IL-8 [28] in normal plasma samples and in intravenous immunoglobulin (IVIg) preparations have been reported [21,22]. Significant levels of high-avidity neutralizing antibodies to IFNα and IFNβ have been shown to be present in human immunoglobulin G (IgG) preparations [29]. Although the biological significance of ACAAs in normal healthy subjects remains unclear, the precise immunological function, whether agonistic or antagonistic will be of interest, particularly the roles of ACAAs in immunoregulation and resolution of inflammatory disease [30,31,32,33]. 

### 2.2. Deleterious Effects of ACAAs 

ACAAs are able to modulate normal immunity and may play a role in the pathogenesis of autoimmune and immune deficiency disease [33,34]. Functional deficiencies of cytokines may result from neutralizing ACAAs inhibiting cytokine signaling, regardless of cytokine concentration, due to the existence of multiple clones of ACAAs. Some examples of the deleterious effects of ACAAs are listed in the ensuing paragraphs. 

Endogenous anti-IFNγ antibodies are associated with infections with tuberculosis, non-tuberculous mycobacteria (NTM), *Cryptococcus neoformans*, *Penicillium marfannei*, and species of non–typhoidal *Salmonella* [34,35]. An estimated 81% of patients with recurrent non-tuberculous mycobacterial infections have high levels of anti-IFNγ neutralizing antibodies, and decreased levels of serum IFNγ. Krisnawati et al. demonstrated that these patients’ serum blocked IFNγ activation of STAT1 and transactivation of IRF1 [36]. Some, but not all anti-IFNγ antibodies bound to a major epitope region (amino acid residues 121–131) required for IFN receptor activation. It is of interest that the patients’ sera cross reacted with the Noc2 protein of *Aspergillus* spp., which shares homology with the epitope [37]. Rituximab and cyclophosphamide have been shown to improve infection by restoring the function of IFNγ in these patients [38,39].

Neutralizing autoantibodies against type I interferons, IL-17 and IL-22 contribute to the development of autoimmune polyendocrinopathy candidiasis ectodermal dystrophy (APECED, also known as type I autoimmune polyendocrinopathy) syndrome, a rare, autosomal recessive disorder caused by mutations in the AIRE gene [32,33]. Chronic mucocutaneous candidiasis (CMC) is associated with anti-interleukin (IL)-17A, anti-IL-17F, or anti-IL-22 autoantibodies [32]. Although high-titer neutralizing autoantibodies to IFN-α and IFN-ω are present in APECED and inhibit the expression of IFN-responsive genes, they do not seem to be associated with increased risk of infection, possibly because of the redundancy of type I IFN species [32]. Neutralizing anti-IL-12p70 autoantibodies were the only identifiable immune defect in a patient with severe recurrent Burkholderia gladioli lymphadenitis [40]. Neutralizing anti-IL-6 antibodies have been described in recurrent episodes of bacterial infections without an increase in C-reactive protein (CRP) level, consistent with impaired IL-6 mediated synthesis of this acute-phase reactant by the liver [41,42]. IL-8 (CXCL8) is a chemokine that is a potent neutrophil chemoattractant and activator. Anti-IL-8: IL-8 complexes exhibit proinflammatory activity, triggering activation and degranulation of neutrophils in the alveolar fluids of patients with acute lung injury [43,44]. 

### 2.3. ACAAs in SLE

ACAAS against type I and II interferons [45,46], G-CSF [47], TNFα [48], IL-1α [49], IL-6 [50], and IL-10 [51] have been described in small patient cohorts in SLE (Table 1).

A recent comprehensive analysis of ACAAs in a large number (498) of rheumatic disease patients included 199 with SLE, 150 with Sjogren’s syndrome (SS), and 149 with RA [52]. Functional analysis as assessed by the inhibition of cytokine-induced signal transduction or protein expression of 24 ACAAs was performed. Thirty-eight percent of SLE, 42% of SS, and 20% of RA patients were positive for at least one ACAA. IL-12-induced STAT-4 phosphorylation was prevented by anti-IL-12 autoantibody positive sera in 10% of SLE patients and 55% of SS patients. IL-22-induced STAT-3 phosphorylation was blocked in a similar fashion to patients with APECED, in only one anti-IL-22 autoantibody sera positive patient with SS, but who did not have candidiasis. Thirty eight percent of the 199 SLE patients had at least one ACAA, 10.5% had two or more, and 7% had four or more. Of interest, these were predominantly autoantibodies against interferons and the interferon-responsive chemokine IP-10. However, only the autoantibodies against type I interferon, IL-12 and IL-22 were neutralizing, as demonstrated by their ability to block cytokine-induced signal transduction or protein expression.

Nearly 50% of SLE patients with anti-type I interferon autoantibodies inhibited the action of their target cytokine, and all were of relatively high titer. Thus, anti-IFNα autoantibodies, particularly those with blocking activity, exhibited effects similar to therapeutic monoclonal anti-INFα in normalizing the expression of type-I IFN inducible genes. Interestingly, the presence of anti-interferon-γ autoantibodies was associated with higher clinical SLE disease activity (as measured by the SLE disease activity index, hypocomplementemia, and elevated titers of double-stranded-DNA antibody), and demonstrated increased expression of IFN-α/β-inducible genes compared to those with anti-IFNα autoantibodies and healthy controls. Thus in this study, autoantibodies to type I and type II interferons in SLE had dissimilar functional roles and cellular effects on disease expression, and differed from those in patients with other anti-cytokine autoantibody-associated immunodeficiencies.

#### 2.3.1. Anti-Interferon Autoantibodies in SLE

SLE was one of the first diseases in which natural autoantibodies against human IFN-α were reported in 1982 [45]. Since then, autoantibodies against type I and II IFNs have been reported in up to 27% of SLE sera [10,46]. As described in the previous section, Gupta et al. found that autoantibodies against type I interferon exhibited neutralizing activity, while autoantibodies to interferon-γ corresponded with greater disease activity, anti-double-stranded-DNA antibodies, and interferon-α/β-inducible gene expression [52].

Using an ELISA specific for anti-IFN IgG antibodies to assess the pharmacokinetics of the anti-human IFN monoclonal antibody rontalizumab in SLE, Morimoto et al. observed anti-interferon antibodies (AIAAs) in 27% of rontalizumab–naïve patients, the majority of whom had significantly lower levels of serum type 1-IFN and downstream IFN pathway activity [10]. The patients could be further divided into IFN^low^ and IFN^high^ based on the level of serum type 1 IFN bioactivity, IFN regulated gene expression, levels of BAFF, anti-ribosomal P and antichromatin antibodies, and AIAA status. Patients with AIAA had lower disease activity compared to IFN^high^ patients, and their sera were effective in neutralizing type 1 IFN activity in vitro. These findings suggest that AIAAs may potentially act as immunoregulators that modify the course of SLE by dampening the effects of IFN. Although their impact on the IFN signature and SLE pathogenesis remains unclear, the potential of AIAAs to influence interferon signaling, disease activity, and response to biologic therapeutics could be substantial.

#### 2.3.2. Anti-BAFF Autoantibodies in SLE

Several recent studies have reported raised levels of endogenous anti-BAFF autoantibodies in SLE both in adult [53,54] as well as in pediatric patients [55]. Raised anti-BAFF autoantibody levels were found to correlate with disease activity in pediatric SLE patients [55]. In contrast, a Swiss study of adult SLE patients did not demonstrate an increase in anti-BAFF autoantibody levels or any association with SLE disease activity while serum BAFF–IgG complexes were associated with serological and clinical SLE disease activity [13]. We recently observed elevated levels of anti-BAFF antibodies in the majority of our multi-ethnic Asian SLE patients, which correlated negatively with clinical disease activity, levels of anti-dsDNA antibodies, and serum BAFF, suggesting that they may be immunomodulatory in nature and may serve as a tool in monitoring disease progression [54].

Some of the explanations proffered for the differences between studies include differences in population patient characteristics, genetic and environmental factors as well as whether the anti-BAFF antibodies are stimulatory or inhibitory. We hypothesize the reduced efficacy of belimumab observed in some SLE patients may be due to ACAAs binding to BAFF close to the epitope recognized by the monoclonal antibody, thereby neutralizing or impairing its action. Functional assessment of BAFF ACAAs would provide further information regarding their biological significance and precise neutralizing or potentiating capacity.

#### 2.3.3. Anti-Chemokine Autoantibodies in SLE

Interferon-γ-inducible protein-10 [IP-10] mediates immune cell trafficking from the circulation to inflamed tissues. As mentioned earlier, the chemokine IP-10 is strongly associated with SLE disease activity and may serve as a potential biomarker for disease flare. Autoantibodies against IP-10 were discovered in SLE for the first time by Gupta et al. [52]. To date, there have been no further studies in SLE regarding this ACAA. Thus, further studies in other populations will be required to determine their biological significance and clinical relevance in SLE.

## 3. Implications for Future Developments in SLE

### 3.1. Assays for ACAAs

As there is wide variation in the methods used to detect ACAAs, efforts to standardize screening methods are needed. As outlined by Meager [22], all current techniques have their advantages and drawbacks. Alteration of the native cytokine in assays is common. Recombinant cytokines used in solid phase assays such as the commonly used ELISAs retain their peptide sequence, but the translational modification is lost, while tagging of the cytokine in fluid assays such as radioimmunoassays also alters the antigen. Functional assays are also required to determine the neutralization ability of the ACAAs. However reference preparations of neutralizing autoantibodies are not readily available, thus there is no uniform measurement of neutralizing activity. 

### 3.2. Development of Therapeutics

#### 3.2.1. Anti-Specific Cytokine Targeted Therapies

The development path of targeted cytokine therapies in SLE has been a difficult one. The anti-BAFF monoclonal antibody belimumab has modest efficacy with approximately a third of patients in most studies demonstrating a reduction in disease activity, its main benefits being the reduction in corticosteroid dose after six months of therapy [57,58,59,60]. However, other cytokine targeted therapies have been disappointing. Agents targeting type 1 IFNs have not met clinical trial endpoints [61]. The anti-IL-6 monoclonal antibody sirukumab did not demonstrate efficacy or an acceptable safety profile [62,63]. While the lack of success may be due to many factors including patient heterogeneity and inadequacy of outcome measures, the presence of preexistent endogenous anticytokine antibodies to the targeted cytokine may also play a role. Screening for the prevalence and neutralizing capability of endogenous ACAAs may be useful in the design of future trials to allow for the selection of patients with a greater likelihood of response. The presence of high titer neutralizing ACAAs may indicate that that individual patient is unlikely to respond to targeting of the same cytokine with an exogenous antibody. This is an area that warrants further investigation given the heavy investments put into the development of each new agent. 

#### 3.2.2. Targeting the JAK/STAT Pathway May Be More Efficacious 

As reviewed in this issue of *Cells* [64], simultaneous suppression of multiple cytokines with JAK inhibitors have shown promising results in Phase II clinical trials [64,65].

#### 3.2.3. Therapy with Cytokines and Cytokine Immunization in SLE

Disturbances in regulatory T cell (Treg) homeostasis from the acquired deficiency of interleukin-2 (IL-2) contribute to SLE pathogenesis [66,67]. Low-dose IL-2 therapy is now being evaluated in clinical trials as it has been shown to restore Treg homeostasis in SLE [68,69,70,71]. Interestingly, there was no difference in the serum levels of IL-2 autoantibodies between responders and non-responders to low dose recombinant IL-2 therapy in one study [56], although the development of treatment induced neutralizing antibodies to IL-2 has been previously reported [72].

IFNα Kinoid (IFN-K) is a therapeutic vaccine composed of IFNα2b coupled to a carrier protein that induces a polyclonal anti-IFNα response that has a broad neutralizing capacity of IFNα subtypes, resulting in decreased IFN- and B cell-associated transcripts [73,74]. Further evaluation in a large placebo-controlled trial is awaited.

#### 3.2.4. Possible Therapies to Avert the Development of SLE 

As cytokine disturbances precede clinical disease in SLE (outlined in Section 1.1), it may be useful to investigate the development of ACAAs during the pre-classification phase of SLE. A more in-depth knowledge of the dynamics of cytokine dysregulation may allow the development of better therapeutic strategies to prevent the development of clinical disease. 

#### 3.2.5. Large Scale Informatics May Improve Therapeutic Approaches 

The difficulties faced in advancing the development of new therapeutics for this complex disease may only be alleviated by the use of big data, a strategy already being employed in the research consortia that have been initiated [75,76]. Without sufficient data on disease biology (e.g., the incorporation of information ACAA and other items), only a minority of patients may demonstrate a response to agents that are targeted at different pathways.

## 4. Conclusions

The cytokine biology of SLE is complex and challenging as the heterogeneity of cytokine dysregulation underlies the heterogeneity of this disease. Knowledge about endogenous ACAAs in SLE is limited and further exploration as to their role in dysregulated cytokine biology is required. A deeper knowledge of the true prevalence and better understanding of the biological significance of preexisting endogenous ACAAs would be beneficial in refining the selection of patients in whom therapeutic anti-cytokine antibody would be the most efficacious. There is also the prospect of earlier intervention to avert the development of clinical SLE disease with a deeper understanding of cytokine dysregulation in pre-clinical disease. As more and more therapeutic anti-cytokine antibodies become available, there is a compelling need to define the role of endogenous ACAAs in the pathogenesis of SLE to identify suitable individual patient subsets with a greater likelihood of response and avoid the implementation of costly therapeutics that might be of little benefit. 

## Figures and Tables

**Table 1:** Anti-cytokine autoantibodies in systemic lupus erythematosus.

Cytokine Autoantibody	%Prevalence in SLE pts (No.)	Correlation with Corresponding Cytokine Levels	Functional Assays	Clinical Associations	References
IFNα	27 (49)			Associated with decreased biological activity of circulating IFNα and lowered disease severity	[10]
	42 (76)			Higher levels in clinically quiescent disease. Clinically quiescent disease demonstrated higher levels of anti-IFNα autoantibodies	[46]
Granulocyte Colony-Stimulating Factor (G-CSF)	50 (32)		A neutralizing effect of anti–GCSF antibodies on its target molecule was found in 3 of the 9 patients tested	Exaggerated serum level of G-CSF, low neutrophil count	[47]
TNF-α		Serum levels of autoantibodies to IL-1, IL-6, IL-10 and TNFα were not significantly correlated with circulating levels of their corresponding cytokines		Significantly lower levels of anti-TNFαantibodies were found at disease exacerbation	[48]
IL-1α	4.7 (64)		Neutralizing		[49]
IL-6	-	No			[50]
IL-10	17.5 (14)	No			[51]
BAFF		Weakly correlated, and with levels of BAFF–IgG complexes			[13]
		No	IgG anti-BAFF autoantibodies in some individuals were capable of blocking BAFF signaling through the BAFF receptor	Associated with a more severe SLE disease profile and elevated IFN signature	[53]
	100 (121)	Negatively correlated with BAFF levels		negatively correlated with clinical disease activity, antidsDNA and BAFF levels	[54]
	62.2 (28)			Correlated with disease activity	[55]
IL-2	18.4 (152)			Higher serum anti-IL-2 IgG present in SLE patients with alopecia	[56]
